# *Lactobacillus* and *Limosilactobacillus* MAGs from alcoholic fermentation in sugarcane biorefineries

**DOI:** 10.1128/mra.00705-25

**Published:** 2026-02-18

**Authors:** Carolina Teixeira Martins, Andreas K. Gombert, Andressa M. Venturini

**Affiliations:** 1 Faculdade de Engenharia de Alimentos, Universidade Estadual de Campinas 28132, Campinas, São Paulo, Brazil; 2 Department of Environmental Science, American University 8363, Washington, DC, USA; California State University San Marcos, San Marcos, California, USA

**Keywords:** metagenomics, lactic acid bacteria, biorefinery, contamination

## Abstract

We recovered and characterized four bacterial MAGs from two Brazilian sugarcane biorefineries, with the aim of investigating the microbial environment during fuel ethanol production. MAGs belonged to *Lactobacillus amylovorus* and *Limosilactobacillus fermentum*, both known lactic acid bacterial contaminants. Genomic analyses revealed key functional traits but no resistance or virulence genes.

## ANNOUNCEMENT

Bioethanol offers environmental benefits over gasoline, and sugarcane-based biorefineries play a pivotal role in national economic development and sustainability (1). Microbial contamination — particularly by lactic acid bacteria — remains a major challenge, decreasing ethanol yields and prompting the use of environmentally unfriendly antimicrobials (2, 3). To investigate the microbial ecology underlying these processes, we sampled two sugarcane biorefineries in Brazil (São Paulo state) during the 2024 harvest.

Samples S222 and S223 were collected from biorefineries UIRA (tank 8; −22.585626, −47.529090) in mid-season (April 2024, 3–4 months after harvest began) and USM (tank 5; −21.319242, −48.117107) at the end of the season (November 2024, 1 month before harvest ended), respectively. From each tank, 12 mL of fermentation mash was collected into 15-mL sterile tubes containing 30% glycerol and stored at −80°C. 1.8 mL of each sample was centrifuged at 10,000 × *g* for 1 min, with the resulting pellet resuspended in 0.9% saline and centrifuged again. DNA was extracted with the DNeasy UltraClean Microbial Kit (Qiagen, Hilden, Germany) and metagenomically sequenced on a NextSeq 2000 (2 × 300 bp) using the Illumina DNA Prep kit (Illumina, San Diego, CA, USA) at NGS Soluções Genômicas (Piracicaba, Brazil). BCL Convert v2.6.2 (Illumina) was used to remove adapters and convert raw data to FASTQ files.

Sequences were processed on KBase (4) with default parameters unless stated otherwise. Reads (16.7 M for S222 and 15.6 M for S223) were quality-controlled using FastQC v0.12.1 (5) and Trimmomatic v0.36 (adapters, TruSeq3-PE-2; sliding window minimum quality, 20; head crop length, 10; and minimum read length, 50) (6). Remaining reads (15.7 M for S222 and 14.6 M for S223) were assembled separately using MEGAHIT v1.2.9 (preset, meta-sensitive) (7) and binned using CONCOCT v1.1 (minimum contig length, 2,000) (8). Low-quality metagenome-assembled genomes (MAGs) were removed using CheckM v1.0.18 (reference tree, full tree; completeness, ≥ 50%; and contamination, ≤ 10%) (9). MAGs had their relative abundance estimated using Bowtie2 v2.3.2 (preset, very sensitive; maximum fragment length for paired-end alignments, 1,000) (10) and were classified using GTDB-Tk v2.3.2 (R08-RS214) (11), FastANI v.0.1.3 (12), and, outside of KBase (4), TYGS (13). MAGs were annotated using DRAM v.0.1.2 (14) and, within Galaxy v.24.2 (15), screened for plasmids, virulence genes, and antimicrobial resistance genes using ABRicate v.1.0.1 (16), with PlasmidFinder (17), VFDB (18), and the NCBI Bacterial Antimicrobial Resistance Reference Gene Database (19) (updated on 15 December 2024). DRAM-based figures were generated with ggplot2 4.0.0 (20) in R 4.4.1 (21).

We obtained four bacterial MAGs: two *Limosilactobacillus fermentum* (Bin013_S222, Bin097_S223; p_Bacillota, c_Bacilli, o_Lactobacillales, f_Lactobacillaceae, formerly *Lactobacillus cellobiosus*) and two *Lactobacillus amylovorus* (Bin049_S222, Bin074_S223; p_Bacillota, c_Bacilli, o_Lactobacillales, f_Lactobacillaceae) (Table 1), with average nucleotide identity (ANI) between 97.5% and 97.9% relative to their closest references, according to GTDB-Tk (11). Digital DNA-DNA hybridization from TYGS (13) also indicated species-level similarity (d4 ≥ 70%) (22) to type strains: Bin049_S222 and Bin074_S223 to *L. amylovorus* DSM 20531 (80.1% and 80.9%), and Bin013_S222 and Bin097_S223 to *L. cellobiosus* DSM 20055 (91.8% and 88.8%). Notably, d4 values for *L. amylovorus* MAGs relative to DSM 20531 lie near the commonly used subspecies threshold (79–80%) (22). ANI comparisons between the two MAGs of each species (average of 99.5% for *L. amylovorus* and 99.3% for *L. fermentum*) suggest they may represent distinct strains (see reference 23).

**TABLE 1:** Characteristics of each MAG obtained using CheckM v1.0.18 (9)

Bin	Completeness (%)	Contamination (%)	Genome size (bp)	No. of contigs	Longest contig (bp)	*N*_50_ (bp)	No. of ambiguous bases	GC (%)	Coding density (%)	No. of predicted genes	MAG abundance within the sample (%)	Accession number
Bin.013_S222	93.9	0	1,687,395	259	41,677	7,969	0	52.7	87.7	1,856	0.3	JBQWNC000000000
Bin.097_S223	98.6	0	1,868,204	78	120,815	34,237	0	52.5	87.0	1,887	0.6	JBQWMZ000000000
Bin.049_S222	97.5	0	2,089,451	135	85,915	24,573	0	37.9	87.1	2,199	6.6	JBQWNB000000000
Bin.074_S223	97.5	0	1,852,632	92	68,764	25,001	0	38.2	88.2	1,903	19.0	JBQWNA000000000

**Fig 1:**
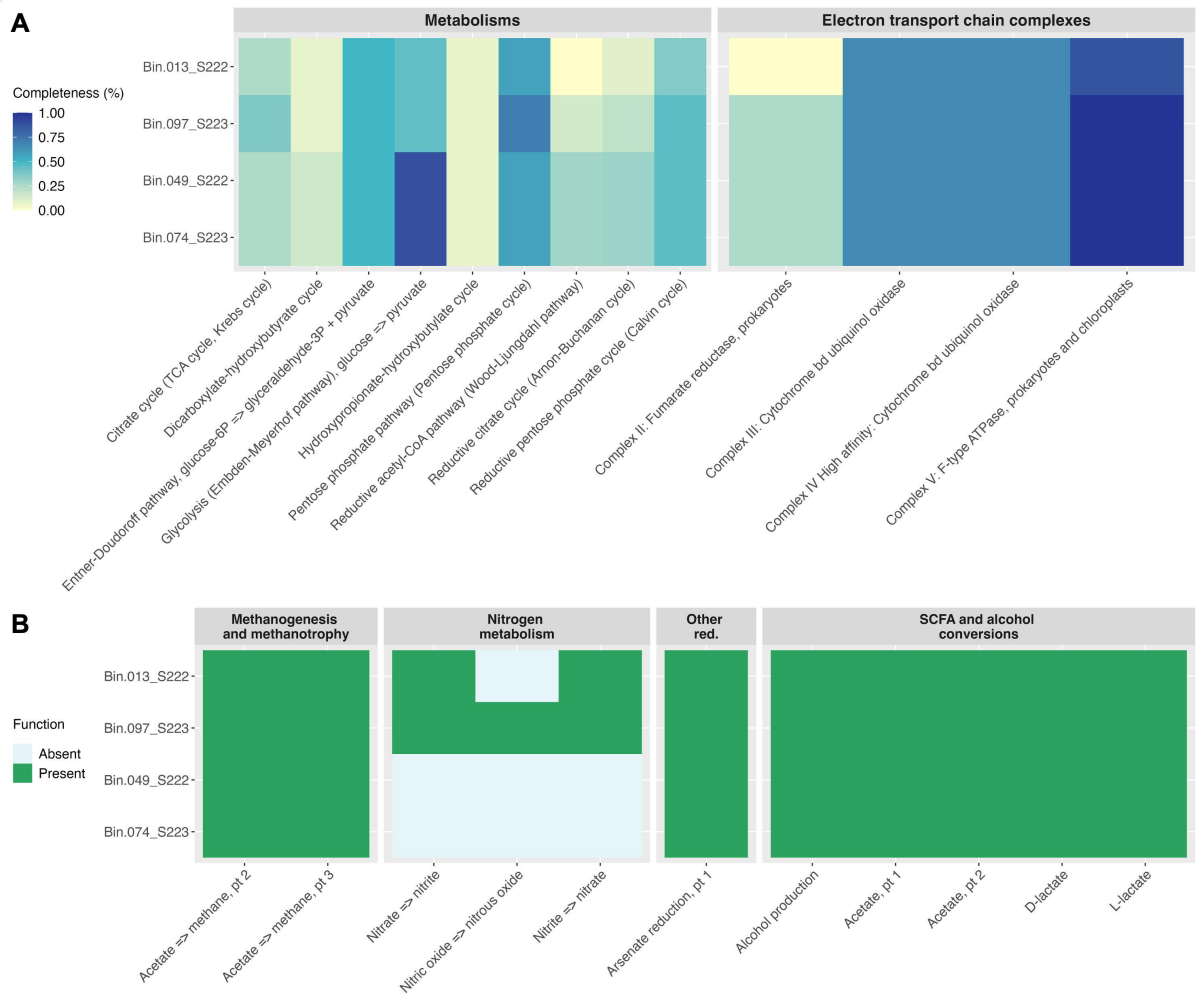
DRAM (14) annotations for each MAG. (**A**) Completeness of relevant metabolic pathways and electron transport chain complexes. (**B**) Presence/absence of relevant metabolic functions. Heatmaps were created based on DRAM output figures and only depict modules that were present in at least one MAG.

DRAM annotations (14) detected 5S rRNA genes and tRNAs in all MAGs, which exhibited variable metabolic potential and electron transport chain completeness (Fig. 1). ABRicate results (16) suggested the presence of plasmid-derived sequences (rep38, 94.2% coverage, 88.9% identity) in Bin049_S222, but no virulence- or resistance-associated genes were detected.

## Data Availability

The sequences of the metagenomes and the metagenome-assembled genomes (MAGs) are available on the KBase platform at https://doi.org/10.25982/206696.195/2570522 and at NCBI under the umbrella project PRJNA1283155 (metagenomes: SRR34283267 for sample S222 and SRR34283266 for sample S223; MAGs: JBQWNC000000000 for Bin.013_S222, JBQWNB000000000 for Bin.049_S222, JBQWNA000000000 for Bin.074_S223, and JBQWMZ000000000 for Bin.097_S223).
